# Psychiatric vulnerability and the risk for unintended pregnancies, a systematic review and meta-analysis

**DOI:** 10.1186/s12884-022-04452-1

**Published:** 2022-02-25

**Authors:** N. N. Schonewille, N. Rijkers, A. Berenschot, J. G. Lijmer, O. A. van den Heuvel, B. F. P. Broekman

**Affiliations:** 1 Department Psychiatry and Medical Psychology, OLVG, Oosterpark 9, 1091 AC Amsterdam, Netherlands; 2 Medical Library, OLVG, Oosterpark 9, 1091 AC Amsterdam, Netherlands; 3 Department Psychiatry and Department Anatomy & Neuroscience, Vrije Universiteit Amsterdam, Amsterdam UMC, Boelelaan 1118, 1081 HV Amsterdam, Netherlands; 4 Department Psychiatry, Vrije Universiteit Amsterdam, Amsterdam UMC, Boelelaan 1118, 1081 HV Amsterdam, Netherlands

**Keywords:** Unintended pregnancy, Perinatal psychiatry, Psychiatry, Mental health, Pregnancy intention, Family planning, Reproductive health, Sexual risk behavior

## Abstract

**Background:**

Unintended pregnancies (UPs) are a global health problem as they contribute to adverse maternal and offspring outcomes, which underscores the need for prevention. As psychiatric vulnerability has previously been linked to sexual risk behavior, planning capacities and compliance with contraception methods, we aim to explore whether it is a risk factor for UPs.

**Methods:**

Electronic databases were searched in November 2020. All articles in English language with data on women with age ≥ 18 with a psychiatric diagnosis at time of conception and reported pregnancy intention were included, irrespective of obstetric outcome (fetal loss, livebirth, or abortion). Studies on women with intellectual disabilities were excluded. We used the National Institutes of Health tool for assessment of bias in individual studies and the Grading of Recommendations Assessment, Development and Evaluation method for assessment of quality of the primary outcome.

**Findings:**

Eleven studies reporting on psychiatric vulnerability and UPs were included. The participants of these studies were diagnosed with mood, anxiety, psychotic, substance use, conduct and eating disorders. The studies that have been conducted show that women with a psychiatric vulnerability (*n* = 2650) have an overall higher risk of UPs compared to women without a psychiatric vulnerability (*n* = 16,031) (OR 1.34, CI 1.08–1.67) and an overall weighed prevalence of UPs of 65% (CI 0.43–0.82) (*n* = 3881).

**Interpretation:**

Studies conducted on psychiatric vulnerability and UPs are sparse and many (common) psychiatric vulnerabilities have not yet been studied in relation to UPs. The quality of the included studies was rated fair to poor due to difficulties with measuring the outcome pregnancy intention (use of various methods of assessment and use of retrospective study designs with risk of bias) and absence of a control group in most of the studies. The findings suggest an increased risk of UPs in women with psychiatric vulnerability. As UPs have important consequences for mother and child, discussing family planning in women with psychiatric vulnerabilities is of utmost importance.

**Supplementary Information:**

The online version contains supplementary material available at 10.1186/s12884-022-04452-1.

## Background

Unintended pregnancies (UPs) are a global health problem of large scale. Every year, 120 million UPs (accounting for 48% of all pregnancies) occur worldwide, although UPs rates differ amongst geographic regions with generally higher rates of UPs in developing countries [1]. UPs could either be mistimed (wanted but not planned at this specific moment in life) or unwanted (not intended at this point nor in the future). UPs are known to have serious consequences as they contribute to adverse maternal and offspring outcomes [2], such as antenatal and chronic depression in mothers [3–7], adverse birth outcomes [2, 8], lower rates of breastfeeding [9, 10], lower quality of mother- and father child interaction [11], and higher prevalence of externalizing problems in puberty in offspring [12]. In addition to adverse effects of unintended births, UPs can also lead to abortions, which are often performed unsafely and account for 7.9% of all maternal deaths worldwide [1, 13]. To prevent UPs, studies investigating risk factors are of utmost importance. Although several risk factors have been identified, such as young maternal age, low educational level (of both parents), and being unmarried [14–18], other potential risk factors, such as mental health, are less explored. Studies already demonstrated that in teenage women with psychiatric conditions (depression, psychosis, and personality disorders) UPs are common [19], but if this also applies for adult women is yet unclear. A previous review on (awareness of) reproductive health problems in women with serious mental illness (that included studies up to 2008) described that the risk of sexually transmitted diseases, pregnancy loss and having more lifetime sex partners is high amongst women with psychiatric conditions [20]. However, unwanted pregnancies and abortions in women who previously reported a psychiatric vulnerability were not the focus of this review. It has been suggested that psychiatric vulnerability (a history of psychiatric disorders according to Diagnostic and Statistical Manual of Mental Disorders (DSM)-IV or 5 and International Statistical Classification of Diseases and Related Health Problems (ICD)-10/11 and/or current psychiatric disorder according to DSM-IV or 5 and ICD-10/11) could influence important factors related to UPs, such as sexual behavior, including victimization of sexual violence [21] or disruption of menstrual cycles due to stress, use of antipsychotic drugs or weight loss in eating disorders [22, 23]. Also, advanced planning capacities, which are required for adequate use of contraceptive methods and family planning, [23, 24] has shown to be diminished in women with psychiatric vulnerability. Thus, we aimed to explore whether psychiatric vulnerability is a risk factor for UPs, by quantifying the presence of UPs amongst adult women with psychiatric vulnerability, in addition to comparing UPs in women with and without psychiatric vulnerability by means of a systematic literature search and meta-analysis.

## Methods

A review protocol was developed based on the Preferred Reporting Items for Systematic Reviews and Meta-Analysis (PRISMA) statement [25] and was registered with Prospero (review number CRD42020221072).

### Information sources and search strategy

The electronic databases PubMed, Embase/Ovid, PsycINFO, Cochrane and Web of Science/Clarivate Analytics were searched on November 6, 2020 (see Additional file 1 for search strategy) to identify studies reporting the proportions of UPs in adult women with (and without) psychiatric vulnerability via self-report, structured clinical interviews, or diagnosis performed by a professional.

There were no restrictions in publication date applied to the search. Only articles in English language were included. Unpublished studies and abstracts were excluded from the review.

### Eligibility criteria

Presence of psychiatric vulnerability at the time of conception was a prerequisite for inclusion. Also, the main outcome, namely UPs that can result in both ongoing pregnancies and elective (induced) abortions, had to be reported. Studies that evaluated pregnancy planning (planned and unplanned pregnancies) instead of pregnancy intention were also included. Studies with or without ‘control groups’ (women without a psychiatric vulnerability) were included.

### Study selection

Studies were eligible for inclusion if the following criteria were met:

- study participants were women who had become pregnant.
- participants were adults: 1) age ≥ 18 years, 2) 95% of the participants was ≥18 years old (mean age − 2 standard deviations ≥18), or 3) a subgroup analysis in women ≥18 years was performed.
- participants had a psychiatric vulnerability (a history of psychiatric disorders according to DSM-IV or 5 and ICD-10/11 and/or current psychiatric disorder according to DSM-IV or 5 and ICD-10/11) via self-report, structured clinical interviews, or diagnosis performed by a professional.
- studies evaluated proportions of unintended, mistimed, unwanted or unplanned pregnancies resulting in ongoing pregnancies or induced abortions.

When articles reported unclear in- and exclusion criteria, the authors were contacted to provide this information. In addition, we contacted authors of studies from 01 to 01-2000 and more recent and invited them to share data in case this was not available for the meta-analysis in published papers.

### Data extraction

Two independent reviewers (NS and NR) screened the identified articles separately based on title and abstract using Rayyan QCRI software [26]. Subsequently, full text screening was performed independently by NS and NR to see whether the articles fulfilled all inclusion and exclusion criteria. If no agreement was reached, a third reviewer (BB) resolved conflicts. Data synthesis was performed by use of a custom-made form that entailed all information necessary to compare studies. Variables analyzed in this review were authors and year of publication, presence and type of psychiatric disorder, presence and type of comparison group (if available), study design, sample size, age of participants, timing and tool used to measure UPs and prevalence of UPs in the study population. NS conducted the full data extraction and NR verified this.

### Assessment of risk of bias

The Grading of Recommendations Assessment, Development and Evaluation (GRADE) [27] method was used to assess quality of the outcome UP. The National Institute of Health (NIH) tools for quality assessment [28] were used to assess the risk of bias in individual studies according to study type. Studies were qualified as ‘good’, ‘fair’ or ‘poor’ considering the risk of bias in that study for our specific outcome ‘UPs’. Hence, studies were assessed solely on the ability to report data on the outcome of interest in this review. Inconsistency was evaluated according to the following levels of heterogeneity by use of I^2^ tests: 25% was considered low, 50% moderate and 75% substantial heterogeneity [29]. A cut-off *p*-value of < 0.05 was used to determine statistical significance of the test. Indirectness was based on the ability of the data to relate to UP rates and imprecision was based on the confidence intervals of the presented results. Publication bias was assessed by evaluating a funnel plot for possible asymmetry. Also, we considered the absence of (un) published articles (with negative findings) in this field. The quality assessments were performed by two individual reviewers (NS and NR), and a third reviewer was involved to resolve conflicts (BB).

### Procedure for data synthesis

Odds ratios (ORs), relative risks (RRs) and risk differences (RDs) were reported if present. In case of observational studies without comparative designs, percentages and means were reported. A meta-analysis of prevalence of UPs amongst women with psychiatric vulnerability was conducted by use of random effects models with the software programmes OpenMetaAnalyst [30] and Rstudio [31]. An I^2^ test was performed to investigate heterogeneity of the studies in addition to sensitivity analyses to control for robustness of the findings [29]. A *p*-value of < 0.05 was considered statistically significant. Separate meta-analyses (forest plots) of specific psychiatric disorder groups were performed in case of ≥4 studies per disorder.

## Results

### Study selection

**Fig. 1:**
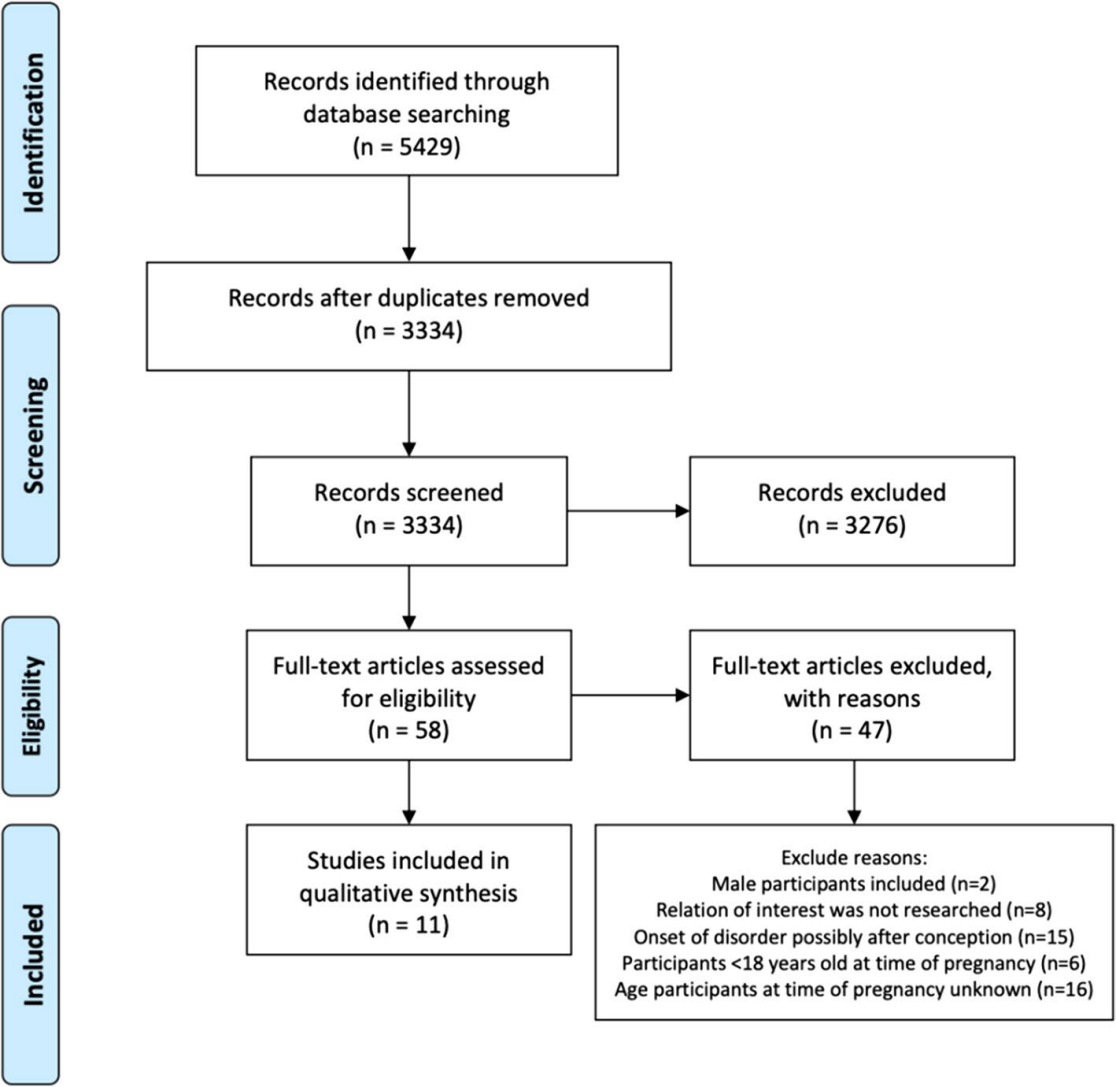
Flowchart of Inclusion process

The inclusion process is displayed in Fig. 1. After electronic searches were performed 5429 articles were extracted and consequently transferred to Rayyan QCRI software [26]. After duplicate removal, screening of title and abstract of 3334 articles was conducted. This resulted in full text reading of 58 articles to assess whether inclusion and/or exclusion criteria were met. Based on the eligibility criteria, eleven articles could be included in the qualitative synthesis. Of the eleven articles, eight articles could be included in the meta-analysis on the prevalence of UPs amongst women with psychiatric vulnerability (Fig. 3) and four studies in the meta-analysis of OR on UPs between women with and without psychiatric vulnerability (Fig. 4).

### Study characteristics

The characteristics and results of individual studies are presented in Table 1. An overall sample of 18,681 women with (*n* = 2650) and without (*n* = 16,031) psychiatric vulnerability were included. Seven categories of psychiatric disorders are represented in this review: eating disorders [32, 42], mood disorders (depression or bipolar disorder) [33, 38, 44, 47, 49], anxiety disorders [44, 47, 49], trauma-related disorders [44, 49], psychosis and related disorders [33, 35], substance use disorders [40, 46, 49], and conduct disorders [43]. Two studies reported on abortion as an outcome of UPs [35, 43] and the other nine studies on (live) births. All studies were conducted in high income countries. Some of the included studies inquired for pregnancy intention during pregnancy, however these studies varied in timing of assessment [32, 40, 44, 47]. Other studies did not report in which trimester women were asked about pregnancy intention [33, 42, 46, 49]. One prospective cohort study assessed pregnancy intention prior to conception and evaluated the number of positive pregnancy tests over the course of one year [38]. In case a woman (without pregnancy aspirations at baseline) became pregnant within twelve months, the pregnancy was defined unintended. In addition, some studies made use of (validated) tools to assess pregnancy intention, while others only reported the questions that were asked to inquire for pregnancy intention. The interpretation of participants’ responses varied: some studies discriminated between unwanted pregnancies and UPs, other studies solely asked for pregnancy planning or pregnancy intendedness. Most studies investigated UPs in women from all ages (within the reproductive phase of life), although one study included only young women (18–20 years) in particular [38].

**Table 1:** Characteristics of the included studies

**First author, year of publication**	**Country**	**Included participants (age range or mean)**	**Study design**	**Measurement of psychiatric disorder**	**Unintended pregnancy assessment**	**Results**
Easter et al., 2011 [32]	UK	171 women with anorexia nervosa (AN), 199 women with bulimia nervosa (BN), 82 women with both AN and BN in addition to 10,636 women without psychiatric disorders. Mean age varied per group (minimum 28.2 years, SD 4.8 and maximum 29.2 years, SD 4.6)	Prospective cohort study, cross-sectional analysis of predictor and outcome of interest	At 12 weeks of gestation, women were asked if they had any history of recent or past psychiatric problems including AN or BN.	At 18 weeks’ gestation, women were asked if their current pregnancy was intentional	UPs in AN group versus general group without psychiatric disorders: OR = 2.0 (95% CI 1.4–2.5, *p* < 0.001)
Green et al., 2008 [33]	UK	39 pregnant women or up to 6 weeks postpartum, with a history of psychotic illness or who were at risk for postpartum psychosis, aged 19–40 years (mean 29.8)	Prospective cohort study with cross-sectional analysis of data on outcome of interest, audit forms were assessed.	Screening for psychiatric disorders by a flowchart at the midwifery pregnancy intake [34]. After referral to a specialist clinic, the presence of a psychiatric condition was reported according to the International Classification of Diseases, ICD-10 by a clinician	Assessment of pregnancy planning was not reported. Intention was assessed during pregnancy up to 6 weeks postpartum	85% UPs in total study population (*n* = 39)
Gupta et al., 2019 [35]	Canada	1565 women, aged 18–49, with schizophrenia or schizoaffective disorder or psychotic disorder not otherwise specified were compared to 36.065 controls	Retrospective cohort study	Validated algorithm requiring one hospitalization or at least two outpatient visits with a diagnosis (based on DSM-IV criteria and ICD-10 codes) of schizophrenia, schizoaffective disorder or psychotic disorder not otherwise specified in the two-year period prior to the index birth [36]	Induced abortions were assessed in patient files, captured by in the ICES datasets [37]	Relative risk of abortion in schizophrenia group versus no schizophrenia group: RR 1.07, 95% CI 0.81–1.42)
Hall et al., 2014 [38]	US	940 women, aged 18–20 year with a strong wish to avoid pregnancy, filled in questionnaires for one year weekly to assess subsequent pregnancies for one year.	Prospective cohort study	Moderate/severe depressive symptoms were assessed at baseline, by use of the CESD-5 scale with a cut-off of ≥4 [39]	Any self-reported pregnancy, after initial wish to avoid pregnancy was defined as UP, assessment was performed weekly	UPs in depressive symptoms group versus control group: OR 12 months =1.2 (CI 0.7–1.9)
Heil et al., 2011 [40]	US	946 pregnant opioid using women aged 18–41	Randomized controlled trial, cross-sectional analysis of data on pregnancy intention	Participants had opioid-abusing disorder according to 1) the Structured Clinical Interview for DSM-IV (First, 1996) or 2) a history of opioid dependence and be at risk for relapse based on their participation in a drug use programs and opioid-positive urine sample before inclusion	Pregnancy intention was assessed by a single question at 6–30 weeks of pregnancy, based on The Pregnancy Risk Assessment Monitoring System (PRAMS) [41]	A total of 817 women out of 946 (86%) report UPs; namely the pregnancy was unwanted (*n* = 252, 27%), mistimed (*n* = 323, 34%) or ambivalent (*n* = 242, 26%).
Micali et al., 2014 [42]	Netherlands	170 pregnant women with lifetime AN, 265 with lifetime BN and 130 with lifetime AN+BN were included, in addition to 1396 pregnant women with other psychiatric disorders and 4367 pregnant women without any psychiatric disorder. Mean age was 29.8–30.2 years, corresponding SD 5.4–5.3)	Prospective cohort study, cross-sectional analysis of data on pregnancy intention	Diagnosis of any psychiatric disorder was assessed by self-report through a questionnaire at 20 weeks’ gestational age	Upon enrolment (during pregnancy) participants were asked about pregnancy intention by a single question	UPs in AN versus control group: OR 1.8 (CI 1.2–2.6, *P* ≤ 0.01) UPs in BN versus control group: 1.2 (0.9–1.7, *P*-value not reported). UPs in AN+BN versus control group: OR 1.5 (CI 1.0–2.4 P ≤ 0.01) UPs in other psychiatric disorders versus control group: OR 1.4 (CI 1.2–1.7, *P* ≤ 0.001)
Pedersen et al., 2011 [43]	Norway	769 girls with mean age 15 years with and without ≥7 conduct disorder symptoms were followed until 20–28 years to assess abortion rate	Prospective cohort study with follow-up period from 1992 to 2005	Number of conduct disorder symptoms was measured by DSM-II-R criteria. A cut off of ≥7 symptoms was used to define ‘severe’ conduct disorder symptoms	Participants were asked about history and number of abortions	Of the women with ≥7 CD symptoms (*n* = 42, psychiatric disorder group), 28.6% had an abortion at age 20–28. Of the women with low CD symptoms (< 7) (*n* = 445, control group), 6.5% had an abortion at age 20–28.
Roca et al., 2013 [44]	Spain	132 women aged 18–46 with selective serotonin reuptake inhibitor (SSRI) use were included (61 with anxiety disorders and 71 with mood disorders)	Prospective cohort study, cross-sectional analysis of data on pregnancy intention	Depression and anxiety disorders were measured with the Structural Clinical Interview for DSM-IV [45]	Pregnancy planning was assessed before 20 weeks of pregnancy by a single question	48.4% of women with depression had UPs, 46% of women with anxiety disorders had UPs.
Tabi et al., 2020 [46]	US	25 opioid addicted pregnant women, aged 20–36, were included	Retrospective cohort study	DSM-V diagnosis at intake in pregnancy addiction program	Assessment not mentioned	100% of women had UPs, 100% had wanted pregnancies
Takahashi et al., 2012 [47]	Japan	Pregnant women, aged 17–44 years, mean age at inclusion 30.5 years and two mothers were < 18 years old	Cross-sectional analysis of pregnancy intention in prospective cohort data	Past and current history of psychiatric disorders were evaluated at study inclusion and confirmed by trained physicians by use of the Structured Clinical Interview for DSM-IV Axis I Disorders (SCID-I) (First, 1996)	National Survey of Family Growth [48] was used to collect information on pregnancy intention, assessed between 14 and 26 weeks of gestation	UPs in mood disorders group versus no mood disorders group: OR 2.05 (CI 1.26–3.33). UPs in anxiety disorders group versus no anxiety disorders group: OR 5.02 (2.19–11.50). UPs in any psychiatric condition group versus no psychiatric condition group: OR 2.10 (1.26–3.26)
Tenkku et al., 2009 [49]	US	484 pregnant women aged 20–39 years were included and assessed for psychiatric disorders. A total of 56 women with anxiety disorders, 67 women with mood disorders and 54 women with substance use disorders were included (some participants reported more than one disorder) and 344 women without psychiatric disorders	Case-control study	Anxiety disorder, mood disorder, substance use disorder and any (other) psychiatric disorders were assessed by use of the Diagnostic Interview Schedule, Version IV [50]	UP was assessed during pregnancy by a multiple choice question, from the 1999 version of the PRAMS [41]	UPs anxiety disorders group versus no anxiety disorders group: OR = 0.60 (CI 0.34–1.08). UPs mood disorders group versus no mood disorders group: OR = 0.65 (CI 0.38–1.12). UPs substance use versus no substance use group: OR = 1.12 (CI 0.59–2.14). UPs any psychiatric condition versus control group: OR 0.83 (CI 0.54–1.28)

### Results per subgroup of psychiatric disorder

The results of all individual studies are presented in Table 1.

#### UPs in women with a psychiatric disorder versus no psychiatric disorder

Three studies compared women with a psychiatric disorder (not specified) to a control group [42, 47, 49]. Tenkku et al. found no difference in OR of UPs between women with and without any psychiatric disorder [49], while both Micali et al. and Takahashi et al. reported higher ORs in women with a psychiatric condition compared to controls [42, 47].

#### Mood disorders

We found five studies that included women with mood disorders [33, 38, 44, 47, 49]. Hall et al. found similar rates of UPs in young women with and without depressive symptoms in a prospective setting even as Tenkku et al. in cross-sectional analyses [38, 49]. In contrast, Takahashi et al. found a higher OR of UPs in women with mood disorders compared to women without mood disorders [47]. Two studies without control groups reported prevalences of UPs (85% in Green et al. and 46–48.4% in Roca et al.) [33, 44].

#### Anxiety disorders

Women with various anxiety disorders were included in three studies [44, 47, 49]. Tenkku et al. showed no difference between women with and without anxiety disorder according to DSM-IV (of which most women had a trauma-related disorder) in UPs [49]. However, Takahashi et al. presented an increased OR of UPs in women with anxiety disorders compared to women without anxiety disorders [47]. In the study sample of Roca et al., 40 women with panic disorder, 16 with generalized anxiety disorder, 10 with obsessive-compulsive disorder, three with post-traumatic stress disorder and two with anxiety disorder not otherwise specified were included, of which 33 had UPs (46% of women with any type of anxiety disorder) [44].

#### Psychosis and related disorders

Women with psychosis and related disorders were investigated in two papers [33, 35]. Green et al. described 85% UPs in 39 women with a risk for postpartum psychotic episode (history of psychotic episode, history of postpartum depression or bipolar disorder) who were in care at a perinatal mental health service during their pregnancy [33]. Gupta et al. compared the incidence of abortions between women with and without schizophrenia in the first year after a previous pregnancy (these pregnancies are referred to as ‘rapid repeat pregnancies’) [35] and found similar rates of induced abortions in both groups.

#### Substance use disorders

Pregnancy intention was assessed in 1455 women who used substances. UPs were often reported in this group of women (74–100%) [40, 46, 49]. Multi-drug use was reported in one study (Tabi et al.) as aside from opioid use, participants reported the (ab)use of cannabis, cocaine, benzodiazepines, methamphetamine, and alcohol [46]. Tenkku et al. assessed nicotine dependence, alcohol and drug abuse in 484 women [49], of which 74% had UPs. Heil et al. found 86% UPs in 946 pregnant opioid addicted women [40].

#### Conduct disorders

One study showed higher rates of (lifetime) abortions in women with a history of high CD symptoms at age 15, (≥7 problems based on DSM-III-R) compared to women with low CD symptoms at age 15. After adjusting for multiple social and psychological confounders, the associations between CD symptoms and abortions remained significant [43].

#### Eating disorders

Assessment of pregnancy intention was performed amongst 927 women with eating disorders in two European studies [32, 42]. In women with anorexia nervosa (AN), OR for UPs were higher than in women without anorexia nervosa, however in women with and without bulimia nervosa (BN), OR for UPs did not differ.

### Risk of bias of included studies

**Fig. 2:**
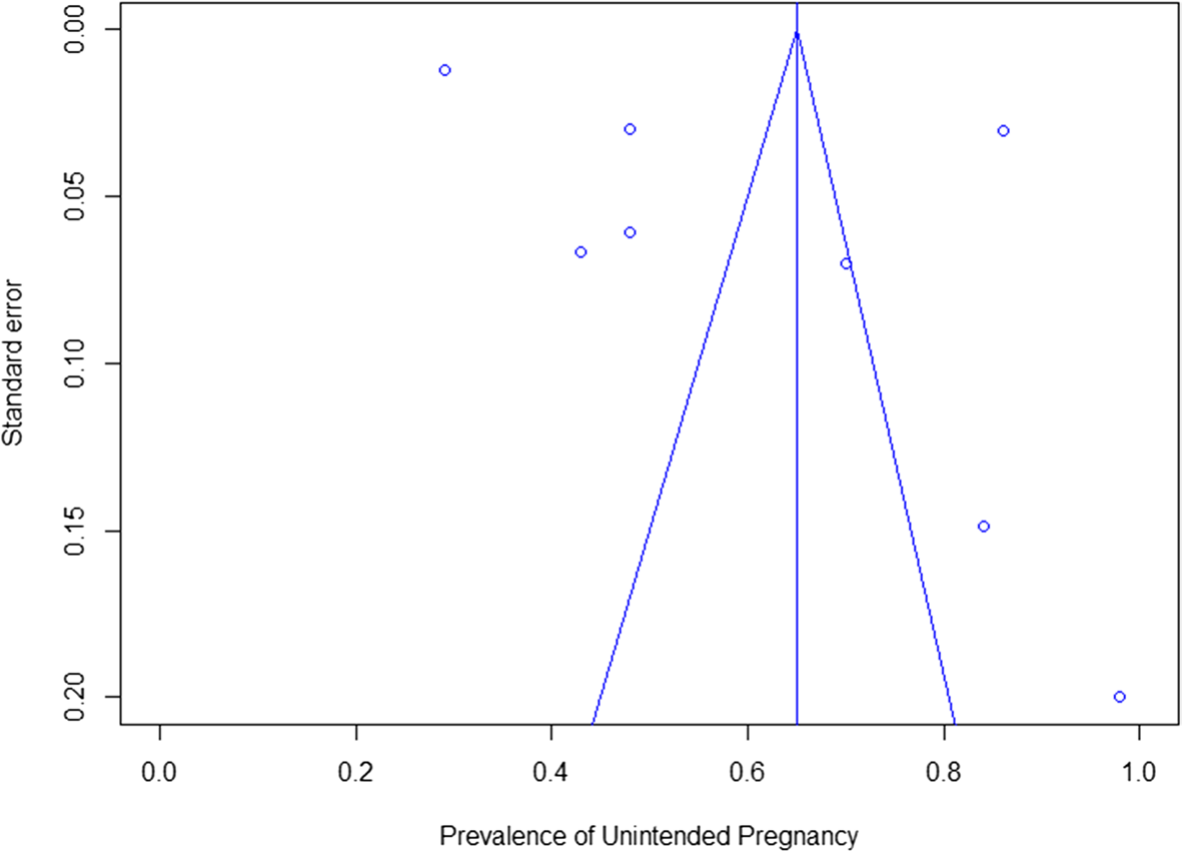
Funnel Plot for studies reporting prevalences of unintended pregnancies in women with psychiatric vulnerability

Quality of the included studies is displayed in Table 2. The outcome UPs graded with the NIH tool [28] resulted in a fair quality for nine out of eleven studies and poor quality in two out of eleven studies. Degree of author agreement was 84% between two reviewers (NS and NR), consensus was reached with a third reviewer (BB). Additional file 2 displays the grading per item in the NIH tool. Risk of bias was high due to cross-sectional analyses of cohort data. Solely one study assessed pregnancy intention in a prospective manner [38], one other study assessed abortion in a prospective manner [43]. In most studies, time from exposure (psychiatric vulnerability) to outcome (UPs) was not measured and/or reported. In addition, UPs were not measured using validated tools. We found that 8 studies primarily focused on UPs or abortions, while three studies included pregnancy intention as secondary outcome or demographic feature [33, 35, 46]. Most studies considered relevant confounders, although small sample sizes limited ability to perform multiple regression analyses in some studies [44, 46]. Most studies had a sample size of less than 600 women, while two studies had a larger sample size: Micali et al. included 1961 women and Heil et al. included 946 women [40, 42]. A funnel plot (Fig. 2) demonstrates the variety in sample sizes and effect sizes per study.

**Table 2:** Quality assessment of included studies

Outcome	Risk of bias assessment	Inconsistency	Indirectness	Imprecision	Publication bias	Overall assessment
*Unintended pregnancy*	Studies had poor to fair quality assessment by use of the NIH tool as displayed in Additional file 2	I^2^ tests showed moderate heterogeneity between studies as the random effects model is accompanied by an I^2^ of 67%, *p* = 0.03	Although the outcome abortion is used as a proxy for UPs in two of the included studies, the outcome of interest (UPs) is investigated directly in all the other studies	As displayed in the random effects model (Fig. 3), the absolute prevalence of UPs amongst women with psychiatric vulnerability is higher than national averages. And although the 95% confidence interval is wide, it is higher than the general population even at the lower border	Most studies were sponsored by national health programs, solely one study was sponsored by industry [47], namely Pfizer Health Research fund. Articles written in non-English language were excluded, which may have led to an overrepresentation of studies from developed countries, causing publication bias. A funnel plot (Fig. 2) shows variety in sample sizes and effect sizes, underscoring the possibility of publication bias	⊗⊗○○ Observational studies start at low certainty, with all 5 domains this does not change (downgrade for inconsistency, upgrade for large effect)

### Data synthesis

**Fig. 3:**
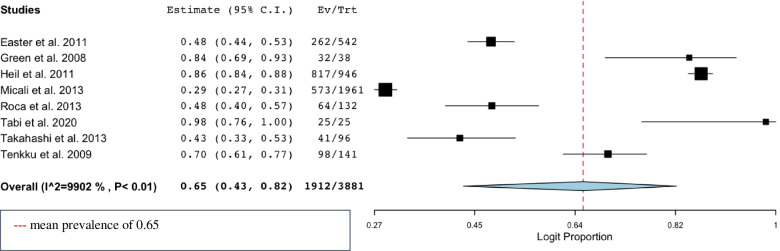
Meta-analyses of prevalence of unintended pregnancies in women with psychiatric vulnerability

**Fig. 4:**
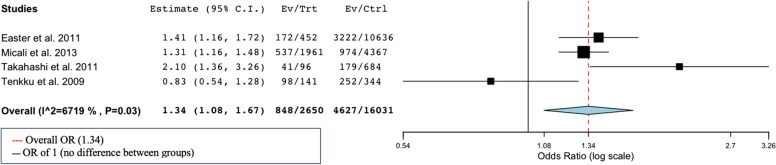
Meta-analysis of OR of unintended pregnancy between women with and without psychiatric vulnerability

A meta-analysis was performed with a random effects model of the eight studies that provided prevalences of UPs amongst 3881 pregnant women (in case studies presented unwanted and unplanned pregnancies instead of UPs, we calculated number of UPs for this meta-analysis) (Fig. 3). We performed a logit transformation of the results, to consider the maximum prevalence of UPs in studies of 100%. Overall, the rate of UPs was 65% (CI 0.43–0.82). Sensitivity analyses were performed and showed that the effect size remained within 95% CI if any of the studies was left out. Moderate heterogeneity was found within the studies as the I^2^ of 67% displays (*p* = 0.03) (see Fig. 3). In addition, separate analyses were performed on the four studies that reported OR of UPs comparing a psychiatric vulnerable group to a control group (Fig. 4). One study on women with eating disorders [32] and three studies on women with a variety of psychiatric vulnerabilities (mood disorders, anxiety disorders, eating disorders, substance use disorders and/or psychosis) [42, 47, 49]. The overall odds of UPs were higher in women with psychiatric vulnerability compared to women without psychiatric vulnerability (OR 1.34, CI 1.08–1.67), *n* = 18,681.

## Conclusions

### Principal findings

This systematic review shows that studies on UPs in women with psychiatric vulnerability are sparse, and for many relevant psychiatric disorders (such as personality disorders, autism spectrum disorder and trauma related disorders) the risk for UPs remains unknown. However, the studies that have been conducted suggest that psychiatric vulnerability is a risk factor for UPs: women with a psychiatric vulnerability have an overall higher risk of UPs compared to women without a psychiatric vulnerability (OR 1.34, CI 1.08–1.67) and an overall weighed prevalence of UPs of 65% (CI 0.43–0.82). As most studies have explored UPs leading to (live) births and did not include or explore UPs leading to abortions, it is likely that this overall prevalence of UPs is even underestimated.

### Comparison with existing literature

Several mechanisms have been proposed to explain the relation between psychiatric vulnerability and UPs. Planning capacities, perception of risks related to unprotected intercourse and subsequent ability to prevent UPs by use of contraception, even as compliance with contraception methods could be impaired by decreased cognitive or emotional functioning during active (severe) mental disorders like mood disorders, schizophrenia or related psychotic conditions [23, 38, 51, 52]. Manic symptoms in women with bipolar disorder could lead to impulsivity and hypersexuality, resulting in risky sexual behavior [53]. In eating disorders there are a few other mechanisms that should also be considered: oligomenorrhea is common and can be misinterpreted as a lower risk of pregnancy or even beliefs about infertility, which could subsequently lead to unintended pregnancies in case of unexpected ovulation. Also, oral contraceptives will not provide prevention of UPs in case of (frequent) purging [22, 42]. Moreover, previous data suggest that in women who requested a termination of pregnancy, traumatic experiences such as sexual violence were prevalent, even as depression and anxiety symptoms [54].

Unfortunately, the extent to which women in the studies included in this review were facing active and/or severe psychiatric symptoms at time of conception was not always clearly described. Some authors, like Micali et al., separately analyzed women with symptoms in the year prior to their pregnancy and found they were more prone to UPs than women with a history of psychiatric disorders [42]. Based on available data in our review, we were not able to conclude whether this finding applies to all psychiatric diagnostic categories.

### Gaps in literature

Although we included studies covering a variety of psychiatric disorders, we conclude that studies on common psychiatric disorders like personality disorders, attention deficit hyperactivity disorder and autism spectrum disorder are lacking. Further studies are needed to investigate UPs in women with these disorders.

Although several studies included women with mood and anxiety disorders, absolute numbers of participants were small. As mood and anxiety disorders are known to be the most prevalent mental disorders, that are almost twice as common in women than in men, it is especially important to understand the role of these disorders in relation to UPs, hence further studies in this field should also be encouraged [55–57]. As none of the studies included in this review were conducted in low-income countries our findings may not apply for low-income countries. Several studies have described that UP rates are similarly high or even higher in low-income countries compared to high-income countries [1, 58, 59], and that the adverse effects of UPs in low-income countries are severe [60].

### Strengths and limitations

Our review has several strengths. First, the extensive search in electronic databases that included all psychiatric disorders, allowed us to gain insight in various specific psychiatric disorders in relation to UPs in addition to an overview of the overall presence of psychiatric vulnerability in relation to UPs. Moreover, we accepted both ongoing pregnancies and induced abortions as outcomes of UPs as previous studies underscored the importance of identifying abortions in women with psychiatric conditions as elective abortions can be a result of UPs [20, 61].

However, our review also has several limitations. We only included studies that were written in English language which may reduce generalizability, however, peer-reviewed studies in other languages were relatively rare. Also, the studies included in the review had fair to poor quality ratings for the primary outcome, used varying psychiatric disorders as control group within studies, used various methods to assess the outcome pregnancy intention (by live births or abortions), differed in timing of measurement of pregnancy intention (which is key in preventing recall bias [62]), and showed divergent results. Pregnancy intention was only measured with validated tools in a few studies [40, 47, 49], while most studies used a single question which may lack nuance [32, 38, 42, 44], or the way of measuring was not reported at all [33, 46]. Abortion was in one study self-reported and in another based on a large obstetric dataset which included surgical abortion registrations [35, 43]. In addition, important confounders such as age, educational level and environmental influences were considered in varying degrees [18]. In particular partner violence and poor partner relationship were posed as risk factor for UPs previously [63] and in women with psychiatric vulnerability, reproductive coercion appears to be common [64, 65]. Lastly, our meta-analysis was limited to only four studies with comparison groups and the overall low quality of this body of evidence limited our capacity to draw definitive conclusions.

### Research recommendations

Ideally, assessment of pregnancy intention is performed 1) by means of a validated tool, and 2) as early in pregnancy as possible. At the same time, prospective settings are time-consuming and might overestimate UP rates since pregnancy intention can change over time [62]. However, prospective designs ensure that psychiatric vulnerability was present before the onset of the UPs, which could give insight in the causality between psychiatric vulnerability and UPS and limit recall bias. Regarding psychiatric vulnerability, we conclude that the onset, duration, and severity of psychiatric vulnerability are important to include, to understand the relation between psychiatric vulnerability and UPs. Last, we recommend that relevant confounders like race, household income, marital status, age, partner relationship, partner violence and reproductive coercion are also taken into account when investigating UPs.

### Implications

In conclusion, we have found a high prevalence of UPs in women with psychiatric vulnerability, and an increased risk of UPs in psychiatric vulnerable pregnant women compared to pregnant women without psychiatric vulnerability. Given the known adverse outcomes of UPs for maternal and offspring health, we underline the importance of discussing family planning with all women at reproductive age with psychiatric vulnerability routinely to avoid any harm due to UPs.

## Supplementary Information

**Additional file 1.** Search strategy electronic database.

**Additional file 2.** Quality assessment of included studies according to Quality assessment tools by National Institutes of Health (2014).

## Data Availability

Data sharing is not applicable to this article as no datasets were generated or analysed during the current study but published previously in studies included in this systematic review and meta-analysis.

## Abbreviations

UP: Unintended pregnancy
DSM: Diagnostic and Statistical Manual of Mental Disorders
ICD: International Statistical Classification of Diseases and Related Health Problems
NIH: National Institute of Health
ORs: Odds ratios
RRs: Relative risks
RDs: Risk differences
AN: Anorexia nervosa
BN: Bulimia nervosa
