# Supplementary material for: Psychiatric vulnerability and the risk for unintended pregnancies, a systematic review and meta-analysis
Source: BMC Pregnancy Childbirth. 2022 Feb 25;22:153. doi: 10.1186/s12884-022-04452-1 (PMC8876535; doi:10.1186/s12884-022-04452-1)
Supplement: Supplementary file 1 — Additional file 1. Search strategy electronic database. [file 12884_2022_4452_MOESM1_ESM.docx]

**Additional file 1 Search strategy electronic database**

Additional file 1 displays the search strategy in electronic database PubMed.

The electronic search as it was performed in PubMed: (Pregnancy, Unplanned[Mesh] OR ((unwanted[tiab] OR unmindful[tiab] OR subconscious[tiab] OR undeliberate[tiab] OR deliberate[tiab] OR inadvertent[tiab] OR advertent[tiab] OR planned[tiab] OR unplanned[tiab] OR unintended[tiab] OR intended[tiab] OR mistimed[tiab] OR mistiming[tiab]) AND (pregnancy[MeSH] OR pregnan*[tiab]))) AND (Mental Disorders[MeSH] OR Psychiatric Diagnosis[MeSH] OR Mental Health[MeSH] OR psychiatric[tiab] OR mental health[tiab] OR anxiety[tiab] OR agoraphobi[tiab] OR compulsi*[tiab] OR panic[tiab] OR phobi*[tiab] OR hypochondri*[tiab] OR bipolar[tiab] OR bi-polar[tiab] OR mood[tiab] OR cyclothymic[tiab] OR depress*[tiab] OR dysthymi*[tiab] OR dissociati*[tiab] OR feeding and eating disorder*[tiab] OR feeding disorder*[tiab] OR eating disorder*[tiab] OR food intake disorder*[tiab] OR anorexia[tiab] OR binge-eating[tiab] OR bulimia[tiab] OR personality disorder*[tiab] OR antisocial[tiab] OR anti-social[tiab] OR borderline[tiab] OR dependent personalit*[tiab] OR avoidant personalit*[tiab] OR histrionic[tiab] OR paranoid[tiab] OR schizoid[tiab] OR schizotypal[tiab] OR narcissistic[tiab] OR affective disorder*[tiab] OR psychotic[tiab] OR psychosis[tiab] OR delusional disorder*[tiab] OR schizophren*[tiab] OR schizoaffective[tiab] OR schizo-affective[tiab] OR insomni* OR substance-related disorder*[tiab] OR alcohol-related Disorder*[tiab] OR amphetamine*[tiab] OR cocaine[tiab] OR inhalant abus*[tiab] OR marijuana OR narcotic-related disorder*[tiab] OR neonatal abstinence syndrome[tiab] OR substance abuse[tiab] OR substance withdrawal[tiab] OR tobacco[tiab] OR stress disorder*[tiab] OR trauma related disorder*[tiab] OR stressor related disorder*[tiab] OR adjustment disorder*[tiab] OR posttraumatic stress[tiab] or post-traumatic stress[tiab] OR ptsd[tiab] OR attention deficit and disruptive behavior[tiab] OR somatoform disorder*[tiab] OR body dysmorphi*[tiab] OR conversion disorder*[tiab] OR factitious disorder*[tiab] OR munchausen[tiab] OR munchhausen[tiab]).
